# Comprehensive Identification of Mitochondrial Pseudogenes (NUMTs) in the Human Telomere-to-Telomere Reference Genome

**DOI:** 10.3390/genes14112092

**Published:** 2023-11-17

**Authors:** Yichen Tao, Chengpeng He, Deng Lin, Zhenglong Gu, Weilin Pu

**Affiliations:** 1 MOE Key Laboratory of Contemporary Anthropology, School of Life Sciences, Fudan University, Shanghai 200438, China; yctao19@fudan.edu.cn (Y.T.); dlin21@m.fudan.edu.cn (D.L.); 2 Greater Bay Area Institute of Precision Medicine (Guangzhou), Fudan University, Nansha District, Guangzhou 511458, China; hcp182@126.com

**Keywords:** mitochondrial DNA, NUMTs, human reference genome, ATAC-seq

## Abstract

Practices related to mitochondrial research have long been hindered by the presence of mitochondrial pseudogenes within the nuclear genome (NUMTs). Even though partially assembled human reference genomes like hg38 have included NUMTs compilation, the exhaustive NUMTs within the only complete reference genome (T2T-CHR13) remain unknown. Here, we comprehensively identified the fixed NUMTs within the reference genome using human pan-mitogenome (HPMT) from GeneBank. The inclusion of HPMT serves the purpose of establishing an authentic mitochondrial DNA (mtDNA) mutational spectrum for the identification of NUMTs, distinguishing it from the polymorphic variations found in NUMTs. Using HPMT, we identified approximately 10% of additional NUMTs in three human reference genomes under stricter thresholds. And we also observed an approximate 6% increase in NUMTs in T2T-CHR13 compared to hg38, including NUMTs on the short arms of chromosomes 13, 14, and 15 that were not assembled previously. Furthermore, alignments based on 20-mer from mtDNA suggested the presence of more mtDNA-like short segments within the nuclear genome, which should be avoided for short amplicon or cell free mtDNA detection. Finally, through the assay of transposase-accessible chromatin with high-throughput sequencing (ATAC-seq) on cell lines before and after mtDNA elimination, we concluded that NUMTs have a minimal impact on bulk ATAC-seq, even though 16% of sequencing data originated from mtDNA

## 1. Introduction

Mitochondrial DNA (mtDNA) does not only thrive in the mitochondria of the cell, it also thrives in various bits and pieces within the nuclear DNA of the chromosomes. These are called nuclear mitochondrial DNA segments (NUMTs) or mitochondrial pseudogenes [1,2,3].

NUMTs may either be fixed (most of the alleles annotated in the human genome) or polymorphic in populations (present in some but not all individuals) [4]. A recent study involving 66,083 individuals suggests that more than 99% had at least 1 of 1637 polymorphic NUMTs. This is the most comprehensive report on polymorphic NUMTs to date [5]. The number of NUMTs fixed in the human reference genome depends on the build version. The widely used human reference genome hg19 build encompasses a range of 612 to 1049 NUMTs, with a total length falling between 249,506 and 536,061 base pairs [1,6,7,8]. NUMTs in the more complete assembled hg38 were converted from hg19 using the UCSC Liftover tools, which is not advisable because NUMTs on newly assembled contigs may become lost. Furthermore, of greater concern is that the current human reference (hg38 and hg19) is a mosaic of genomes assembled from over 20 individuals, with approximately 70% of the genome contributed by a single individual [9,10]. The T2T Consortium’s CHR13 stands out as the sole fully assembled human reference to date, with assembly of the remaining 8% of unknown regions in hg38 [9], and it is also the only reference derived from a single individual (except for the Y chromosome). Given that the human T2T-CHR13 DNA assembly is currently the most complete human reference genome, it is of vital importance to understand how many mitochondrial sequences are embedded within it. A recent study has compiled a mitochondrial blacklist for T2T-CHR13, which serves as an exclusion set in ATAC-seq practices [11]. However, it may not adequately represent the compilation of NUMTs.

NUMTs can introduce bias into the results of chromatin-targeting technologies, mtDNA heteroplasmy detection, and mtDNA PCR assays [7,11,12,13,14,15]. To be more specific, sequences are cross-mapped due to NUMTs’ resemblance to genuine mtDNA. Shorter reads or amplicons are more susceptible to the influence of cross-mapping. In the practice of ATAC-seq, up to 80% of mitochondrial sequences may pile up in the NUMT regions [12,16]. The detection of low-frequency mtDNA heteroplasmy is susceptible to the influence of NUMTs, especially in cells with low mitochondrial copy numbers [11,17,18]. Furthermore, a contentious study claims to have discovered evidence for the bipaternal inheritance of mtDNA [19]. However, opponents argue that this could potentially be attributed to the presence of Mega-NUMTs within the genome, which allows PCR amplification and leads to high levels of a mixed haplotype [20,21,22]. On the other hand, NUMTs are not solely associated with negative aspects either, either, as they constitute molecular ‘fossils’ within the more slowly evolving nuclear DNA and are thus (relatively) ‘frozen’ snapshots of ancient mtDNA configuration [3,23]. This trait can be utilized for phylogenetic analysis or for studying archaic introgression [6,23,24,25,26,27,28].

Given the significance of NUMTs, we hold a keen interest in the NUMTs within the first completely assembled human reference genome T2T-CHR13. Currently, several bioinformatics approaches exist for the identification of NUMTs, which can be broadly categorized into reference-based and de novo discovery approaches. An effective approach has been developed which utilizes whole genome sequencing (WGS) to discover novel NUMTs in populations [29]. Reference-based methods rely on the comparison of similarity between mtDNA and the reference genome, utilizing well-known techniques such as BLAST [30] or the Burrows–Wheeler transform [31,32]. However, nearly all comparisons are based on the reference mtDNA sequence—rCRS (revised Cambridge Reference Sequence), which fails to represent the mutational spectrum of mtDNA in populations. In fact, other reference mtDNA, such as the Ugandan Sequence (D38112), can differ from rCRS at up to 90 positions [33], while the Japanese Sequence (AB055387) differs by 50 positions [34]. This implies that a substantial number of NUMTs within the nuclear genome, dissimilar to rCRS, are being overlooked. Some studies have attempted to incorporate known mtDNA mutations into rCRS or generate random mutations through simulation [11,35]. However, these artificial approaches cannot address the challenge posed by the random combinations of the mutations. In theory, there are 4^16,569^ point mutations in the mitochondrial genome, which is an extremely large number, that does even account for indels.

In recent years, the accumulation of human mtDNA sequencing data has enabled us to utilize authentic mtDNA diversity sequences for NUMTs identification. Human mtDNA sequencing data mainly include the full-length sequences of mtDNA and amplified segments targeting the mtDNA control region. Currently, there are sixty thousand complete mtDNA sequences and eighty thousand mtDNA control region sequences available in GenBank. In this study, we downloaded all of these sequences. After excluding similar sequences, an effective human pan-mitogenome was established, encompassing all major mitochondrial haplogroups, thereby representing human mtDNA mutational spectrum. Subsequently, we employed BLAST for longer segments (NUMTs) and Bowtie for shorter segments to identify mtDNA-like segments in the reference genomes such as hg19, hg38, and the newly complete one, T2T-CHRM13. In comparison with the known NUMTs compilation, we identified approximately 10% more NUMTs under stricter thresholds. We propose that this methodology can be applied to the increasingly established pan-genomes in recent years [36,37]. Concurrently, we conducted ATAC-seq on HEK293 cell lines before and after mtDNA elimination. Because of the absence of nucleosome protection around mtDNA, the accumulation of mtDNA fragments in NUMT regions can result in the generation of spurious peaks in ATAC-seq experiments. In our findings, the erroneous pile up of mtDNA sequences within NUMT regions is not substantially severe, with minimal impact on peak calling of ATAC-seq practical applications. We also identified mito-blacklist regions that have potential influence in ATAC-seq. These mtDNA-like sequences (NUMTs, short segments and mito-blacklist) identified in this study can serve as the infrastructure for mitochondrial-related practices.

## 2. Data and Methods

### 2.1. Preparing the Human Pan-Mitogenome (HPMT)

We retrieved human mitochondrial sequences from GeneBank, downloading 62,052 complete mtDNA sequences and 80,841 control region sequences (Supplementary Materials File S1). These data are continuously updated, with the data downloaded up to 21 May 2023. To eliminate identical sequences, we employed a self-blast approach. Initially, we established a blastn database for these 62,052 sequences and performed self-blast. Identical sequences were retained as one, with a minimum sequence length requirement of 16,530 bp. Haplogrep-v2.4.0 was used for haplotype classification, with a quality threshold set at greater than 0.5 [38]. Self-blast employed blastn’s default parameters, removing approximately 20,000 sequences and leaving 47,525 distinct complete mtDNA sequences. As the control region’s sequences vary in length (from 400 to 1598) and exhibit a higher mutation rate, we did not remove duplicate sequences within the control region. This has no impact on the results because identical sequences mapped to the same regions are subsequently merged. The purpose of removing duplicate sequences was to reduce the computing budget.

Considering that mtDNA is a circular molecule, we added 100 base pairs from the starting end to the terminus of mtDNA molecules to enhance mapping efficiency. The 47,525 complete mtDNA, rCRS, and 80,841 control region sequences were combined into a human pan-mitogenome pool (HPMT) for subsequent analysis, totaling 128,367 sequences.

### 2.2. BLAST and Filtering

We ran BLAST-2.14.0+ on the Linux environment [28]. First, we established blastn databases for the reference genomes hg19, hg38, and T2T-CHR13, respectively. Subsequently, we performed blastn queried HPMT in the reference genome databases, with the parameters set as follows: -evalue 10^−4^, -gapopen 5, -gapextend 2, -penalty-3, and -reward 2, following the methodology of previous studies. The -evalue was set to 10^−4^, a relatively stringent parameter, to ensure precision [1,5,6,7].

Results from blastn were filtered using bash script to extract sequences with an identity greater than 63 and e-value less than 10^−4^. The minimum length of NUMTs should be greater than 28 bp. Currently, no study has defined the minimum length of NUMTs, and we estimated that the minimum length of NUMTs should be greater than 28 bp to ensure precision (Results). Overlapping NUMT regions were merged into one. The bedtools-v2.30.0 “merge” command was employed to merge overlapping regions and create a bed format [39]. NUMTs length statistics and filtering were performed using GenomicRanges-1.50.2.

### 2.3. Alignment of Short Segments

Using the sliding window approach, we progressively segmented HPMT into 20-mer segments by sliding base by base, thereby generating single-end sequencing fastq files with a pseudo sequencing quality of 35, corresponding to a 20× sequencing depth. We then aligned these short reads to the human reference genome using Bowtie-1.3.1 with default parameters [32]. The generated sam files were converted to bam files using samtools-1.11, with all reads containing “N” or IUPAC nucleotide encodings (RYSWKM) removed [36]. Sambamba-0.6.6 was used to remove duplicates [40], and the bedtools-v2.30.0 “bam2bed” command was employed to extract the aligned region to the reference genome [39].

### 2.4. Eliminating Mitochondrial Copy Numbers in HEK293 Cell Lines

We followed the approach of Domenico et al. by using the pCW57-MCS1-P2A-MCS2 (Neo) vector to eliminate mitochondrial copy numbers in HEK293 wild-type cells, referred to as “mtDNA-reduction”. The mtDNA-reduction cell line expressed a truncated segment UL12.5 from herpes simplex virus 1 (HSV-1) [41]. This segment specifically localized to the mitochondria and caused elimination in mtDNA copy numbers through cleavage by the mitochondrial nucleases ExoG and EndoG [42,43].

### 2.5. ATAC-Seq Data Analysis

Raw fastq files underwent quality control using fastqc-v0.11.9 [44], adapter and low-quality reads were trimmed using trimmomatic-0.39 [45], and alignment to the reference genome was performed using bwa-0.7.17 mem [31]. Sambamba was employed for duplicate removal, and samtools was used to extract concordant mapped reads with MAPQ > 30, with sequences mapped to mtDNA (NC_012920.1) removed. Peak calling was performed using MACS2--omodel--shift-100--extsize 200--keep-dup all [46]. The R package diffbind-3.8.4 was utilized for differential peak analysis.

### 2.6. Statistics and Visualization

ChromoMap-4.1.1 was used to create chromoMaps, and Circos-0.69-9 was used to generate Circos plots [47]. Unless otherwise specified, all figures and data statistics in this paper were prepared in the R-4.2.3 environment.

## 3. Results

### 3.1. Human Pan-Mitogenome (HPMT)

**Figure 1:**
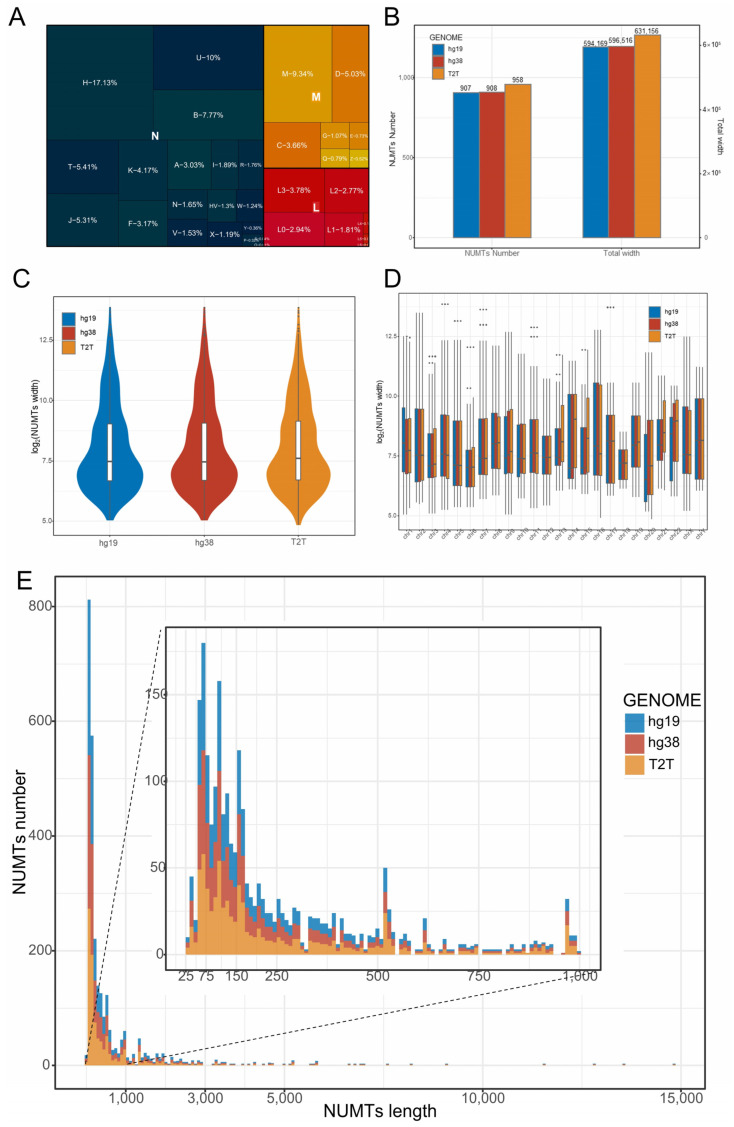
NUMTs in three human reference genomes (hg19, hg38, T2T-CHR13). (**A**) The mitochondrial haplogroups and their proportions in the pan-mitogenome database. The colors represent major haplogroups, and the smaller squares within the same color represent the sub-haplotypes within the major haplogroups. (**B**) The number and length of NUMTs in the three reference genomes. (**C**,**D**) The length (log2) distribution of NUMTs. The colors represent different reference genomes. The points above the box in (**D**) represent outliers in the box plot. (**E**) Histogram of NUMTs length distribution (25 bp~14,855 bp).

We first prepared the Human Pan-mitogenome to represent human mtDNA diversity. We downloaded 62,052 complete human mtDNA sequences and 80,841 mitochondrial control region sequences from GeneBank. After excluding duplicate sequences and filtering out low-quality ones from the complete mtDNA, we obtained a total of 47,525 valid mtDNA sequences. These sequences cover the established major haplogroups, and thus can be considered representative of human mtDNA diversity (Figure 1A and Table S1). Considering the varying lengths of sequences from the mitochondrial control region (ranging from 400 bp to 1598 bp) and the presence of significant variations, we did not remove redundant sequences from the control region. We combined all 80,841 control region sequences with the 47,525 full-length mitochondrial sequences and added the Cambridge reference sequence (rCRS: NC_012920.1) to create the “Human Pan-mitogenome (HPMT)”, comprising a total of 128,367 human mitochondrial-related sequences (Supplementary Materials File S1).

### 3.2. More NUMTs Were Identified Using HPMT

We employed the blastn, utilizing the pan-mitogenome for alignment against reference genomes. The complete mtDNA sequences revealed 814, 815, and 863 NUMTs in the hg19, hg38, and T2T-CHR13, respectively (Table S2). The results excluded contigs other than chromosomes 1 to 22 and the XY chromosome. All identified NUMTs exhibited e-values less than 0.0000908 and sequence identities greater than 65. The three reference genomes have a total length of 584,137 to 620,968 base pairs for NUMTs, with the shortest NUMT being 35 bp and the longest being 14,855 bp, which is nearly equivalent to the length of mtDNA (Table S2). Aligning using control region sequences revealed 313 to 345 NUMTs, all with e-values below 0.0001 and sequence identities greater than 63.37. In aggregate, these NUMTs spanned a length of 89,180 bp to 95,474 bp (Table S2).

We merged the NUMT regions identified from complete mtDNA and the control region sequences. After merging, there were 907, 908, and 958 NUMT regions in the hg19, hg38, and T2T-CHR13, respectively, with corresponding total NUMT lengths of 594,169, 596,516, and 631,156 bp (Table 1 and Figure 1B). This implies that NUMTs constitute approximately 0.02% of the human genome. When comparing the three reference genomes, T2T-CHR13 had more NUMTs than hg19 and hg38, representing 105.6% (958/907) and 105.5% (958/908), respectively. The total length of the CHR13′s NUMTs was also the longest, accounting for 106.2% (631,156/594,169) and 105.8% (631,156/596,516) in comparison to hg19 and hg38, respectively. Furthermore, the median and average length of the identified NUMTs indicated T2T > hg38 > hg19 (Table 1 and Figure 1C–E). These results underscore the influence of genome assembly on NUMT identification, with the highly assembled CHR13 genome revealing more and longer NUMTs, on average.

### 3.3. Comparison with the Known NUMTs Compilation

**Figure 2:**
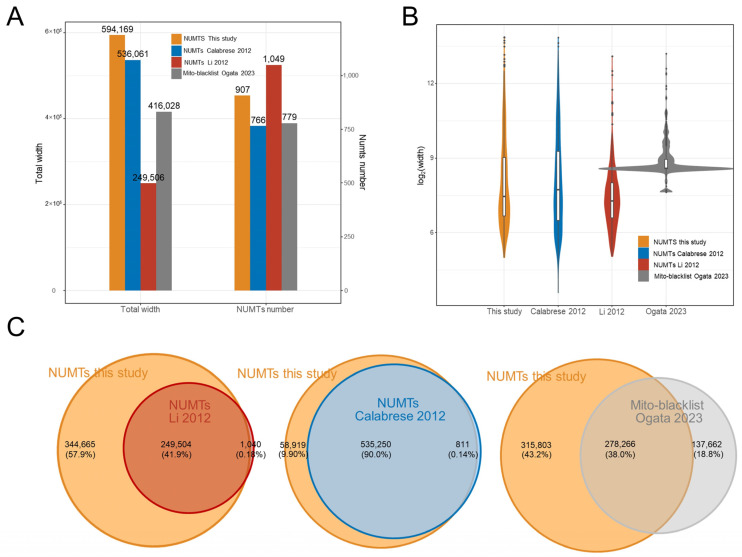
Comparison with the known NUMT compilations (hg19). (**A**) The number and length (log2) of NUMTs identified in this study compared to known NUMTs. (**B**) The distribution of NUMTs length (log2) in different NUMT compilations. (**C**) Overlap between the NUMTs compilation identified in this study and in three previous studies. The union of two NUMT compilations constitutes 100% [7,8,35].

In the reference genome hg19, Li et al. identified 1049 NUMTs with a total length of 249,506 bp [6]. Calabrese et al. also identified 764 NUMTs with a total length of 536,061 bp [5,7]. Here, we compared our NUMTs compilation with their results (Figure 2A,B; Table 1).

As previously mentioned, we identified 907 NUMTs in hg19, totaling 594,169 bp in length. This is 141 more NUMTs and 58,108 bp longer than Calabrese et al.’s results (combining overlapping NUMTs). If we merge Calabrese et al.’s NUMTs with our findings to create a comprehensive set of NUMTs, our identified NUMTs account for 98.6% of the total length (Figure 2C). In the Venn diagram, it can be seen that our NUMTs compilation essentially encompass Calabrese et al.’s compilation, with only a 0.14% loss (Figure 2C). Similarly, our NUMTs compilation also nearly encompasses encompassescompilation, with a loss of only 0.18% of the total length. Because we used the same blastn approach and set a more stringent e-value threshold (10^−4^ compared to 10) than Calabrese et al., theoretically, the number of NUMTs should be lower. The higher quantity of NUMTs can be attributed to the use of the pan-mitogenome. A stricter e-value implies more precise results but also lower sensitivity. An e-value of 10 means that up to 10 hits can be expected to be found just by chance, given the same size of a random database. Considering that HPMT has approximately 10^5^ sequences, multiple hypothesis testing would increase the error rate. To ensure that the blastn results have similar precision to Calabrese et al. without causing excessively low sensitivity, we set the e-value to 10^−4^, aiming to achieve a similar effective e-value (10) as Calabrese et al. (although it may be slightly stricter in reality due to the inclusion of approximately 80,000 sequences in HPMT that are shorter than rCRS). It is worth noting that Li et al. employed a higher “Identity” threshold, resulting in a more “fragmented” set of NUMTs (more in number, but shorter in average length and total length) (Figure 2A–C; Table 1). This suggests that some almost full mitogenome sequences NUMTs (Mega-NUMTs) might have been fragmented into two or more smaller NUMTs. Some studies propose the existence of such Mega-NUMTs in the human genome, so we consider that the NUMTs identified by Li et al. may be somewhat conservative [21,29,48].

Additionally, we compared the overlap of Ogata et al.’s identified mito-blacklist with our NUMTs. The mito-blacklist regions are areas where mtDNA reads erroneously map to the nuclear genome during high-throughput sequencing. These mito-blacklist regions may cause a pile up of mtDNA reads, leading to false peak calling. Typically, mito-blacklist regions partially overlap with NUMTs regions, but they are not entirely identical. In Ogata et al.’s study, 70% (278,266/415,928) of the blacklist region is contained within our NUMTs compilation, but 30% is excluded, and this 30% is from 212 mito-blacklist regions (Figure 2C). These regions may represent mtDNA-like regions in the nuclear genome that are difficult to recognize as NUMTs, such as some shorter segments. This result also suggests that there may be more mtDNA insertions in the nuclear genome than previously known. Similarly, our compilation includes 338 NUMTs that are not part of the mito-blacklist, indicating that not all NUMTs lead to the accumulation of mtDNA reads in the practice of chromatin targeting technologies. This may be related to the mitochondrial haplotype of the samples.

### 3.4. Distribution of NUMTs in the Human Reference Genome

**Figure 3:**
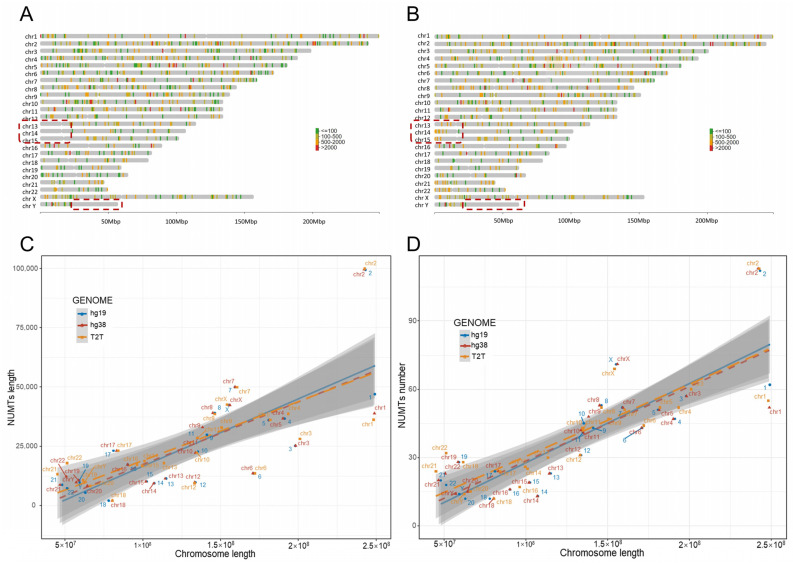
NUMTs on different chromosomes. (**A**,**B**) The distribution of NUMTs on different chromosomes ((**A**) hg38; (**B**) T2T-CHR13). The colors of the bands indicate the NUMTs’ lengths. The red boxes highlight a gap region on the Y chromosome and the short arms of chromosomes chr13, chr14, and chr15. The total length (**C**) and number (**D**) of NUMTs exhibit a linear relationship with chromosome length. Different colors indicate the reference genomes.

**Figure 4:**
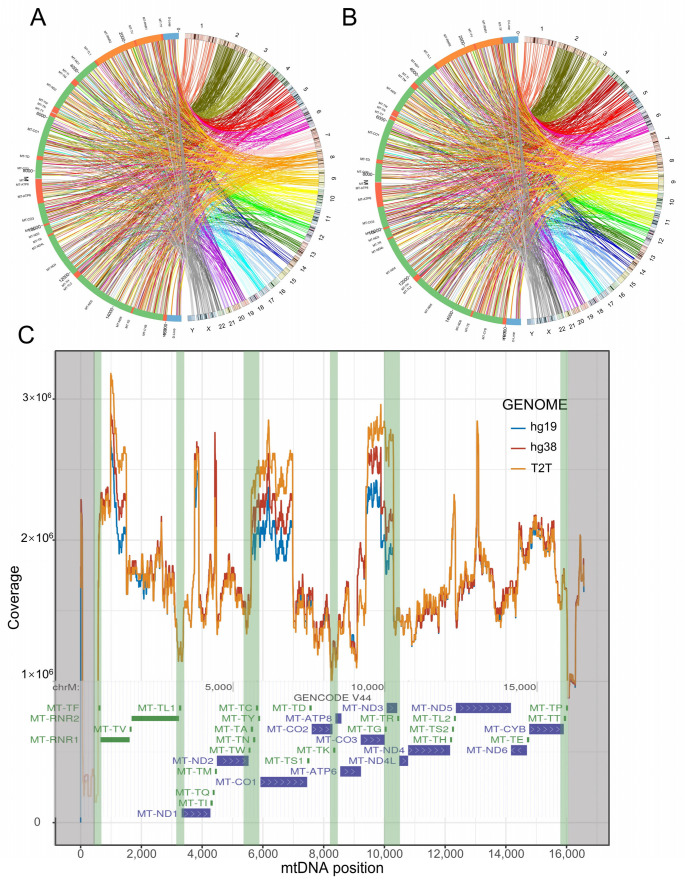
The breakpoints of NUMTs. The Circos plot illustrates the distribution of NUMTs on two reference genomes, hg38 (**A**) and T2T-CHR13 (**B**). The left half-circle in the Circos plot represents the coordinates of the mitochondrial genome, while the right half-circle represents the coordinates of nuclear chromosomes 1–22 and the XY. The connecting lines indicate the breakpoint of NUMTs, and the colors of the lines represent different chromosome origins. (**C**) The coverage of NUMTs mapped to mtDNA. The *x*-axis represents the positions corresponding to mtDNA (1~16,569), while the *y*-axis represents the coverage. Regions with low coverage suggest the presence of breakpoints. The gray and green shading, respectively, highlight breakpoints in the mtDNA control region and tRNA region.

We observed the presence of NUMTs in the short arms of chr13, chr14, and chr15 in the T2T-CHR13 genome. The short arms of these three chromosomes were not fully assembled in hg19 and hg38; thus, there have been no reports of NUMTs in these regions (Figure 3A,B and Figure 4A,B). In general, the distribution of NUMTs on each chromosome is proportional to the chromosome’s length, but chr2 shows a relatively high density of NUMTs (Figure 3A,D). In contrast, chr1, chr3, chr6, and chr12 have relatively sparse NUMTs (Figure 3A,D). Interestingly, there are NUMT-free regions on some chromosomes, with the longest NUMT-free region located on the long arm of the Y chromosome, spanning 35 M in length (Figure 3A,B and Figure 4A,B). This phenomenon is even observed in the fully assembled T2T-chrY and overlaps with the heterochromatic region on the Y chromosome [43].

Mitochondrial DNA coevolves with the nuclear genome, during which mtDNA can break at “breakpoints” and insert into the nuclear genome. We are curious about whether the distribution of breakpoints on mtDNA is uniform. We examined the breakpoints of the 47,525 complete mtDNA and found that the distribution of NUMT breakpoints on mtDNA is not uniform. There are noticeable breakpoints at the boundaries of the mitochondrial control region (16,024~16,569, 1~576). Breakpoints outside the control region mainly originate from the regions containing tRNA genes (Figure 4C). This suggests that the inserted fragments of NUMTs on the nuclear genome primarily originate from the coding genes separated by tRNA genes, with particularly high coverage in MT-RNR1, MT-ND3, and MT-CO1 (Figure 4C). It is intriguing to note that a recent *Cell* paper has reported a novel family of DNA transposons called “tycheposons” in Prochlorococcus bacteria. These transposon elements are similarly cleaved into islands by tRNA, and there is a high degree of overlap between the “breakpoint” regions and tRNA gene regions [49].

### 3.5. mtDNA-like Short Segments within the Nuclear Genome

**Figure 5:**
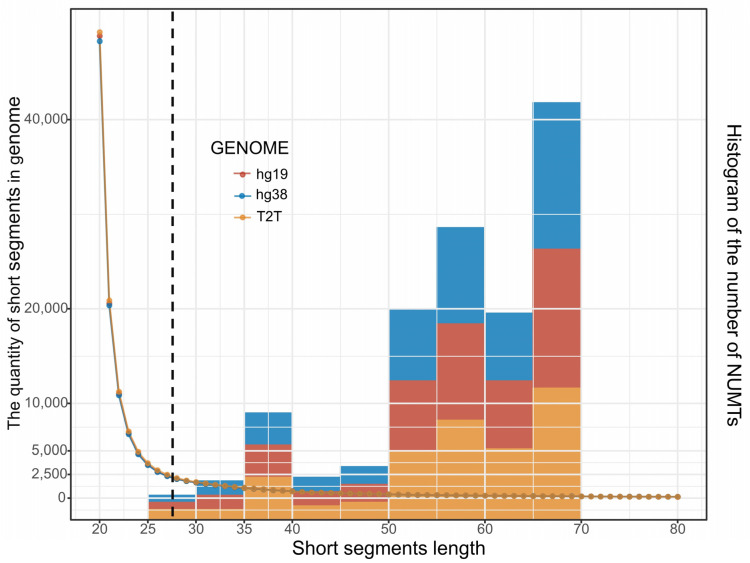
The quantity of 20-mer segments mapped to the nuclear genome. The *x*-axis illustrates the length of mapped positions (mapped by 20-mers). The left *y*-axis corresponds to the number of 20-mer segments, while the right *y*-axis corresponds to the number of NUMTs. The dotted curve illustrates a decreasing trend in the quantity of 20-mers (on the left *y*-axis) as the mapped position length increases. Conversely, the histogram shows an increasing trend in the number of NUMTs as the mapped position length increases. Additionally, it is evident from Figure 1E that shorter NUMTs (<70 bp) are less abundant compared to longer NUMTs (>70 bp). This suggests the potential presence of unnoticed short NUMTs. Additionally, the turning point of the dot curve occurs in the range of 25~30 bp, indicating that we should set the cutoff for the shortest NUMTs in this range (the black dashed line, 28 bp).

Short segments of mtDNA-like regions within the nuclear genome can interfere with short amplicons and cell-free mtDNA detection, and these segments may originate from mtDNA or may result from random nucleotide arrangements. Meanwhile, we observed an absence of short segments within the identified NUMTs (Figure 1E and Figure 5).

Using the slide window approach, we segmented the 128,367 HPMT sequences into 20-mer short segments, referred to as HPMT-20. We discarded segments containing unknown bases ‘N’ and IUPAC nucleotide encodings (RYSWKM). This yielded a total of 854,733,854 short sequences, encompassing 423,658 unique 20-mer types. The most frequently occurring 20-mer appeared 122,764 times, with a median occurrence of 3 times (Supplementary Materials File S2). For 20-mer sequences, there are theoretically 4^20^ possible combinations, amounting to 1,099,511,520 possibilities. However, the human HPMT-20 contains only 423,658 unique sequences, indicating highly compressed and conserved mtDNA sequence information.

Using the Bowtie, which is adept at aligning short sequences, we aligned HPMT-20 to the human reference genome, covering approximately 200,000 nuclear positions with a minimum range of 20 bp and a maximum range of 5862 bp (Table 2). We extracted reads from these positions and found that they originated from roughly 360,000 unique 20-mers, accounting for about 85% of the total 423,658 20-mer types. This suggests that 15% of the short segments in HPMT-20 do not have homology in the nuclear genome. Furthermore, approximately 8000 20-mers have appeared at least three times in the nuclear genome (File S2). We recommend avoiding these highly recurring mtDNA homologous sequences in short amplicons of mtDNA or in cell-free mtDNA detection [13,50].

Additionally, as the length of mtDNA-like regions (mapped by 20-mers) increases, the counts of the 20-mers start to decrease, with a drastic turning point occurring around 25~30 bp (Figure 5). There are only just over 1800 positions exceeding 30 bp in three reference genomes (Table 2). This implies that the minimum segment length for NUMTs should be greater than this range, which is why we have set NUMTs to be greater than 28 bp. The previously identified NUMTs lack short fragment sequences (<70 bp). This could be due to the e-value threshold requirements imposed by the BLAST algorithm. This implies that we may have missed some short fragment NUMTs in the range of 28~70 bp (Figure 1E and Figure 5), suggesting that the actual number of NUMTs in human reference may be greater than initially estimated.

### 3.6. The Impact of NUMTs on ATAC-Seq Appears to Be Limited

In mtDNA, which lacks nucleosome enveloping, the presence of mtDNA fragments piling up on NUMT regions can lead to pseudo peaks in ATAC-seq experiments. These pseudo peaks in NUMT regions should be excluded from the analysis, forming the “Exclude set: mito-blacklist” [14,35]. In this study, we designed experiments to identify mito-blacklist regions and assess their impact in ATAC-seq experiments.

**Figure 6:**
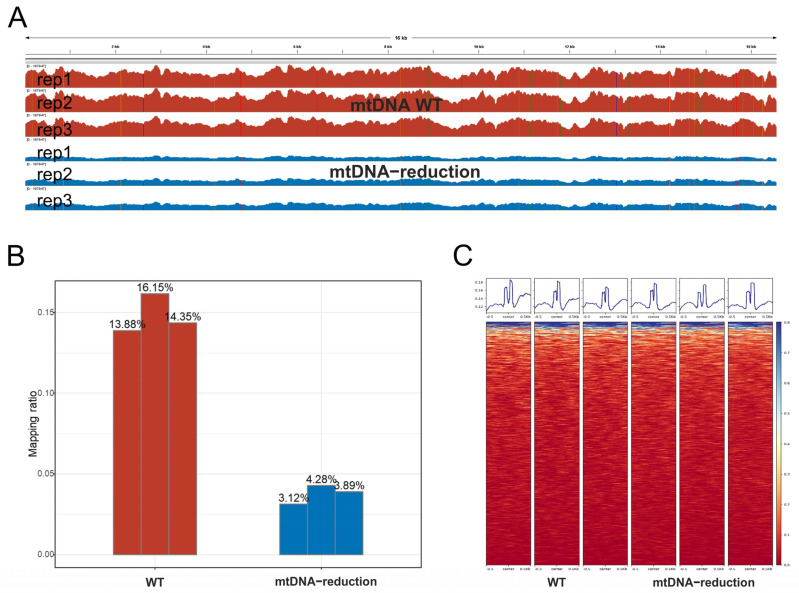
Comparison of ATAC-seq before and after mtDNA reduction. (**A**) Coverage on mtDNA, with red representing mtDNA-WT and blue representing mtDNA-reduction, with three biological replicates for each treatment. (**B**) The proportion of reads aligned to mtDNA in the ATAC-seq sequencing files. (**C**) The peak (top) is consistent in shape between mtDNA-WT and mtDNA-reduction, and there is no obvious accumulation of reads in the heatmap (bottom). This suggests that the impact of NUMTs-Blacklist regions in the nuclear genome on ATAC-seq is minimal.

We initially conducted mtDNA elimination on HEK293 (mtDNA-WT) to generate cell lines with reduced mtDNA copy numbers (mtDNA-reduction). Subsequently, we performed ATAC-seq on both cell lines before and after the mtDNA copy number reduction. The reads proportion derived from the mtDNA-WT replicates ranged from 13.88% to 16.15%, while in the mtDNA-reduction replicates, mtDNA reads accounted for approximately 3.12% to 4.28%, representing an approximate 11% decrease (Figure 6A,B).

We mapped the ATAC-seq reads to the reference genome (T2T-CHR13) to generate BAM files (the compressed Binary representation of sequence Alignment Map). Reads mapped to the mitochondria were removed from the BAM files while retaining the concordant paired align reads mapped to the nucleus. We extracted these concordant reads from the BAM files and aligned them to the HPMT. Successfully aligned reads were considered potential mito-blacklist regions. In the samples of mtDNA-WT and mtDNA-reduction, a range of 2727 to 12,035 reads was aligned to the reference genome (Table S3A), covering positions ranging from 919 to 1735 on the reference genome (Table S3B). We identified a total of 919 mito-blacklist regions on T2T-CHR13, with a length of 251,932 bp (Table S3B). These positions were merged with Ogata et al.’s mito-blacklist and our identified NUMT regions, forming a combined set referred to as NUMTs-Blacklist. NUMTs-Blacklist contained 1595~1978 regions with a total length of 867,093~903,362 bp in three reference genomes (Table 3).

Furthermore, we assessed the impact of NUMTs-Blacklist on ATAC-seq peak calling. Initially, we identified differential peaks between mtDNA-WT and mtDNA-decline samples, revealing a total of 54,613 differential peaks following mtDNA copy number reduction, with 37,914 upregulated and 16,699 downregulated. Only 18 of these peaks were located within NUMTs-Blacklist regions, accounting for a mere 0.33% (18/54,613). Among these, 7 peaks were downregulated, and 11 were upregulated, displaying no preference. We further assessed the peak heights within NUMTs-Blacklist regions and found that the heights of peaks in mtDNA-WT and mtDNA-decline samples were consistent (Figure 6C). In the heatmap of the 1595 NUMTs-Blacklist regions, there was no evidence of reads stacking (Figure 6C). These results suggest that the impact of mitochondrial homologous regions may be limited in ATAC-seq experiments.

In ATAC-seq experiments, even when reads aligned to mtDNA are excluded, a small fraction of reads still align to the NUMTs-Blacklist region. This suggests that NUMTs and mtDNA-like regions can still have an impact on ATAC-seq results, although the impact may be limited. In practice, we recommend merging NUMTs and mito-blacklist as an exclusion set (NUMTs-Blacklist in this study, Table 3).

## 4. Discussion

In comparison to the current reference genome version (hg38), CHR13 contains approximately 6% more NUMTs. Considering that CHR13′s assembly completeness is approximately 8% higher than hg38, this is reasonable. But, it is important to note that CHR13 and hg38 have different sample sources. CHR13 is derived entirely from a grape hydatidiform mole, while hg38 is approximately 70% from a Caucasian individual. Therefore, we cannot rule out individual sample differences as contributing factors. Additionally, we found that breakpoints on mtDNA primarily originate from tRNA and control regions. These regions share the common characteristic of having a relatively high mutation rate, but we cannot currently provide a definitive explanation for the cause of these breakpoints.

We also observed the presence of numerous small segments of mtDNA-like sequences within the nuclear genome, which are too short to be recognized as NUMTs. These “mtDNA-like segments” could either result from mtDNA insertions or arise as coincidental, similar sequences within the vast nuclear genome. Determining their origin solely based on sequence similarity between mtDNA and the nuclear genome is challenging. We can only offer the somewhat arbitrary criterion that segments shorter than 28 bp should not be considered NUMTs, implying that short segments of NUMTs within the nuclear genome might be underestimated.

Through ATAC-seq experiments on cell lines before and after reducing mtDNA copy numbers, we found that the design of the ATAC-seq analysis pipeline can minimize the impact of mtDNA-like regions (NUMTs and mito-blacklist). Specifically, it is advisable to remove mtDNA sequences from the aligned BAM files and select concordant paired-end reads for subsequent peak calling. In our study, mtDNA-like regions affected only approximately 0.33% (18/54,613) of differential peaks. However, considering that mtDNA sequences in the HEK293 cell line used in this experiment only account for 16% of the total reads, the impact may perhaps have been underestimated. It is possible that in cell lines with higher mitochondrial copy numbers, the influence of mtDNA-like regions could be more significant.

## 5. Conclusions

Using the pan-mitogenome (HPMT), we systematically identified mtDNA-like regions within three human reference genomes. This included NUMTs, mtDNA-like short segments, and mito-blacklist regions that may lead to erroneous read stacking in chromatin-targeting technologies. We found that NUMTs may account for approximately 10% more content than previously known, and we reported for the first time the distribution of NUMTs in the only complete human reference genome, T2T-CHR13. Compared to the current reference genome version (hg38), CHR13 contains approximately 6% more NUMTs, including those on the short arms of previously incompletely assembled chromosomes (chr13, chr14, and chr15). The abundance of short segments of mtDNA-like sequences suggests the possible presence of more mtDNA insertion fragments within the nuclear genome. These segments are so short that they are challenging to identify as NUMTs, but they should be considered in the design of short amplicons or in cf-mtDNA detection [13,50]. Through ATAC-seq experiments on cell lines before and after reducing mtDNA copy numbers, we conclude that mtDNA-like regions are unlikely to significantly affect ATAC-seq results, even when 16% of the sequencing data originate from mtDNA.

We provide NUMTs, mtDNA-like short segments, and mito-blacklist files of the three reference genomes (Files S2–S5). This can serve as the infrastructure for mitochondrial-related bioinformatics practices.

## Figures and Tables

**Table 1:** NUMTs compilation in this study and previous studies.

**Previous Studies**	**NUMTs**	**Total Length**	**Min**	**Median**	**Mean**	**Max**	**E-Value_Max**	**Iden_Min**
hg19_2012_Calabrese	766	536,061	12	212	700	14,835	10	63.52
hg19_2012_Li	1049	249,506	33	155	238	8795	0.00005	78.11
**This Study**	**NUMTs**	**Total Length**	**Min**	**Median**	**Mean**	**Max**	**E-Value_Max**	**Iden_Min**
hg19	907	594,169	32	176	655	14,855	0.0001	63.89
hg38	908	596,516	32	176	657	14,855	0.0001	63.89
T2T-CHR13	958	631,156	28	194	659	14,855	0.0001	63.37

**Table 2:** Short mtDNA-like segments (20-mer) within the nuclear genome.

Total Segments	Unique Segments	Reference	Mapped Pos	Total Length	Min	Median	Mean	Max	>30 Pos
854,733,854	423,658	hg19	200,968	4,172,251	20	20	21	5862	1808
		hg38	199,556	4,142,766	20	20	21	5862	1802
		T2T-CHR13	202,859	4,207,476	20	20	21	3420	1872

**Table 3:** Potentially impactful mtDNA-like regions on ATAC-seq (NUMTs-Blacklist).

Reference	Number	Total Length	Min	Median	Mean	Max
hg19	1978	901,808	20	194	456	15,010
hg38	1964	903,362	20	198	460	15,010
T2T-CHR13	1595	867,093	20	301	544	15,010

## Data Availability

This study’s ATAC-seq fastq files and blastn output files have been deposited in ZENODO (https://doi.org/10.5281/zenodo.8383968, accessed on 27 September 2023).

## Supplementary Materials

The following supporting information can be downloaded at: https://www.mdpi.com/article/10.3390/genes14112092/s1, Table S1. Mitochondrial haplogroups in Human Pan-mitogenome (HPMT); Table S2. Statistics of blastn results from complete mtDNA and control region; Table S3. Homologous reads between the nuclear genome and HPMT in ATAC-seq sequencing data. File S1: Accession number of 128,367 sequences in this study; File S2: NUMTs in three reference genomes; File S3: Summary of mtDNA-like short segments; File S4: Mito-blacklist identified using ATAC-seq; File S5: NUMTs-Blacklist.
